# Serum irisin associates with breast cancer to spinal metastasis

**DOI:** 10.1097/MD.0000000000010524

**Published:** 2018-04-27

**Authors:** Zheng-ping Zhang, Xue-fang Zhang, Hui Li, Tuan-jiang Liu, Qin-peng Zhao, Lin-hong Huang, Zi-jun Cao, Li-min He, Ding-jun Hao

**Affiliations:** Department of Orthopedics, Hong Hui Hospital, Xi’an Jiaotong University College of Medicine, Xi’an, China.

**Keywords:** breast cancer, cross-sectional study, metabolic syndrome, serum irisin, spinal metastasis

## Abstract

The aim of this study was to determine whether the serum level of irisin can be a candidate to predict the spinal metastasis in patients with breast cancer.

In a cross-sectional study, 148 patients were recruited. Of those, 53 (35.8%) had spinal metastasis. The baseline characteristics were compared by status of spinal metastasis. Multiple logistic regression analysis was used to determine whether the serum irisin can be a candidate for predicting breast cancer to spinal metastasis. The correlation coefficient analysis was used to confirm the correlation between the serum irisin and lipid metabolic parameters and body mass index (BMI), respectively.

The serum irisin was higher in patients without spinal metastasis (7.60 ± 3.80). Multivariable analysis showed that the serum irisin was protective to the presence of spinal metastasis in patients with breast cancer after adjustments of age and BMI (odds ratio, 0.873; 95% confidence interval, 0.764–0.999). Furthermore, there was a positive correlation between the serum irisin and BMI (*r* = 0.263). The patients with metabolisc syndrome (MetS) had a higher level in serum irisin. In addition, the higher numbers of MetS components were associated with higher serum irisin.

Higher serum irisin can be a protective factor of spinal metastasis in patients with breast cancer. The BMI is positively associated with the serum level of irisin. Furthermore, patients with MetS tended to have a higher level of serum irisin.

## 1 Introduction

Skeleton, especially spine, is one of most frequent sites for breast cancer metastasis.^[1]^ Interestingly, bone often presents as the single site for metastasis in patients with breast cancer,^[1]^ implying it may have special characteristics for facilitating the metastasis of breast cancer cells. Nevertheless, the patients with bone metastasis can easily suffer from the complications caused by cancer-induced bone disease, including pain, fracture, and spinal cord compress, which then distinctly impact the quality of life (QoL) and even cause the poor clinical outcomes.^[2,3]^ It can be useful to identify potential novel biomarkers related to bone metastasis to predict the possibility of bone metastasis in breast cancer patients and to guide the strategy of regimens. In previous researches, Ferreira et al found the higher level of serum YB-1 associated with more severe metastatic state.^[4]^ In addition, in another study, cathepsin V and D were found to be correlated to breast cancer metastasis and the cancer to bone metastasis meant poor prognosis.^[5]^ Besides above, other factors, such as interleukin-17, parathyroid hormone-related protein, and transforming growth factor-β, plastin-3, c-Src, FOXC1, and some metabolites, were also considered to be the candidates to predict bone metastasis in patients with breast cancer.^[4,6–10]^

Irisin is one of novel myokines, which was identified in recent years. The “myokines” are a family of hormones secreted by muscles. They participate in several physiological and pathological processes, for example, lipid metabolism and cancer progression.^[11–13]^ Currently, irisin was found to improve the quality of skeleton^[14]^ and its serum concentration in patients with breast cancer was lower than healthy participants.^[15]^ In addition, there still remains a debate about its role in metabolic syndrome (MetS). The circulating irisin levels were positively associated with the higher body mass index (BMI) or fasting insulin.^[16]^ However, the different roles of serum irisin for MetS were presented in Chinese and White or Black individuals.^[16–19]^

Considering the serum irisin influences both skeleton and breast cancer, we are interested whether the serum level of irisin may be considered as a candidate to predict the spinal metastasis in patients with breast cancer. For our cross-sectional study, we compared the serum level of irisin in the participants with breast cancer with/without spinal metastasis, and also interpreted the triangular correlation between the presence of MetS, the serum irisin, and spinal metastasis in these participants.

## 2 Patients and methods

### 2.1 Subjects

A total of 148 female patients diagnosed with breast cancer were recruited in our medical center between March 15, 2012, and December 31, 2016. Criteria for the exclusion were as follows: recurrence/relapse, diagnosed in other places, received regimens before, insufficient imaging and laboratory test results, had infective disease, received lipid-lowering therapies, and refusal of consent after being provided information about the study. We collected data on demographics, risk factors, blood pressure (BP), serum level of irisin, and laboratory tests associated with lipid metabolism. Ethical approval to conduct the study was obtained from The Ethical Committee of Hong Hui Hospital (Xi’an Jiaotong University College of Medicine, Xi’an). All necessary consents were obtained from all participants.

### 2.2 Definition of spinal metastasis

The spinal metastasis was confirmed by performing examinations of spinal x-ray, bone scanning followed by positron emission tomography-computed tomography and biopsy.

### 2.3 Definition of metabolic syndrome

The MetS was diagnosed under the criteria established by the Adult Treatment Panel III (ATP III), including abdominal obesity, insulin resistance, dyslipidemia, and hypertension.^[20]^ In this study, we selected BMI instead of waist circumference as a criterion because BMI and the presence of MetS have a strong correlation.^[21]^ Patients were recorded as having MetS if they had ≥3 of following: a body mass index (BMI) ≥25 kg/m^2^, high BP (systolic BP ≥130 mmHg and/or a diastolic BP ≥85 mmHg), a high fasting glucose level (≥6.1 mmol/L), a high triglyceride level (≥1.69 mmol/L), and a low level of high-density lipoprotein cholesterol (HDL-C; <1.29 mmol/L for women). All of the anthropometric measurements were collected by trained nurses following standard protocols.

### 2.4 Biochemical measurements

Parameters of lipid metabolism, including serum triglyceride (TG), total cholesterol (TC), low-density lipoprotein cholesterol (LDL-C), high-density lipoprotein cholesterol (HDL-C), apolipoprotein A-I (apo A-I), and apolipoprotein B (apo B), and fasting blood glucose levels were collected (Beckman Coulter, Inc., Brea, CA, USA). All of the blood samples were collected between 6 and 7 am after an overnight fast for >8 hours.

### 2.5 Serum irisin measurement

Serum irisin concentration was measured using the enzyme-linked immunosorbent assay kits (Aviscera Biosciences, Santa Clara, CA); this kit was proven to be efficient in Chinese people.^[17]^ The intra and inter-assay variations were both <10%.

### 2.6 Statistical analysis

All analyses were conducted using SPSS Statistics software (version 21.0; IBM SPSS Inc., Chicago, IL). Data are shown as mean ± standard deviation (SD) or percentages, as appropriate. Differences between groups regarding continuous variables or proportions were compared using the Student *t* test or χ^2^ test, respectively. One-way analysis of variance was used in comparisons among multiple continuous variable groups. A multivariable logistic regression analysis was performed to evaluate the association between the serum irisin and breast cancer to bone metastasis after adjusting for other potential confounders. Results are given here with odds ratios and 95% confidence intervals (CIs). Pearson product moment correlation analysis or Spearman rank correlation analysis was performed to find the correlation among serum irisin, the parameters of lipid metabolism, as appropriate. A *P* value <0.05 was considered statistically significant.

## 3 Results

### 3.1 Participants’ characteristics

Totally, 148 female patients (mean age, 60.58 ± 7.79 years) were enrolled in the study. Among the 148 participants, 53 had spinal metastasis (35.8%). Patients without spinal metastasis were of a significantly higher BMI, whereas no statistical significance was seen in age. For parameters of lipid metabolism, there were significant differences between the 2 groups for serum level of LDL-C and HDL-C. Furthermore, the patients with spinal metastasis were of a significantly lower serum irisin. The total number of patients with MetS was 47 (31.8%); the proportion of those with MetS was significantly higher in patients without spinal metastasis group than in those with spinal metastasis group (MetS, 37.9% vs. 20.8%; *P* = .042), as shown in Table 1.

**Table 1:**
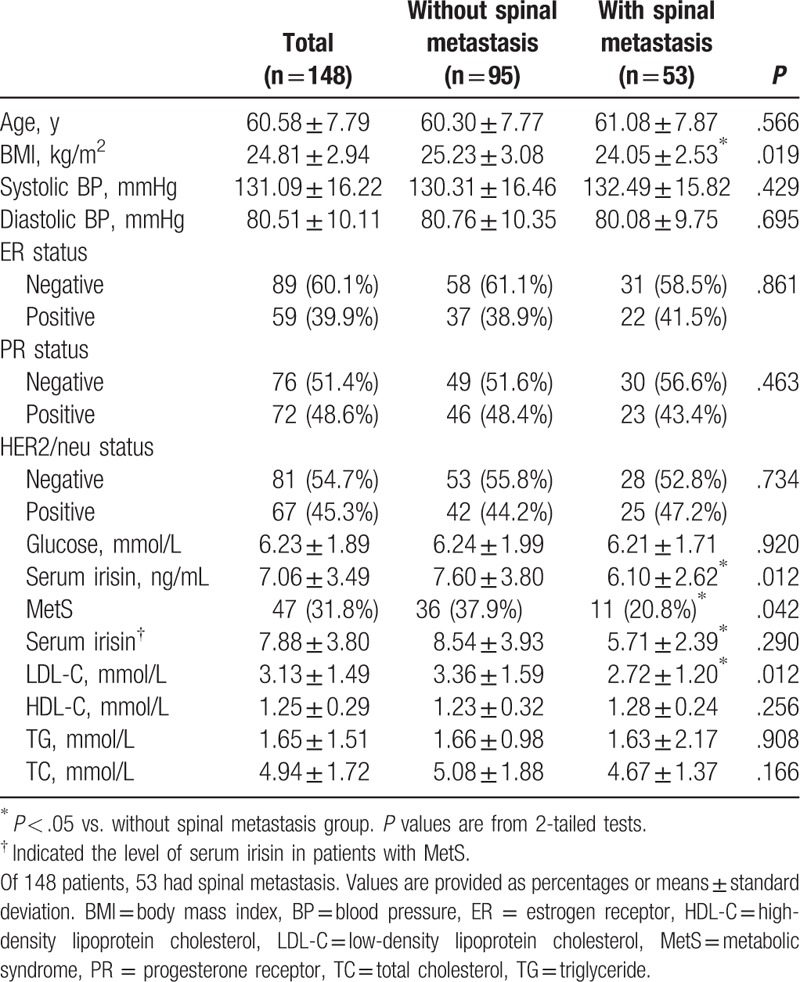
Baseline characteristics.

### 3.2 Association of serum level of irisin with spinal metastasis

Table 2 shows the results of multivariate logistic regression analysis for with/without spinal metastasis. In our model (for spinal metastasis), multivariable analysis showed that the serum level of irisin has a protective role for spinal metastasis in breast cancer patients after adjusted for age and BMI (crude odds ratio [OR], 0.850, [95% confidence interval, CI 0.746–0.968]; adjusted OR, 0.873 [95% CI 0.764–0.999]). Other independent risk factors were LDL-C (crude OR 0.165 [95% CI 0.048–0.569]; adjusted OR, 0.162 [95% CI, 0.045–0.581]).

**Table 2:**
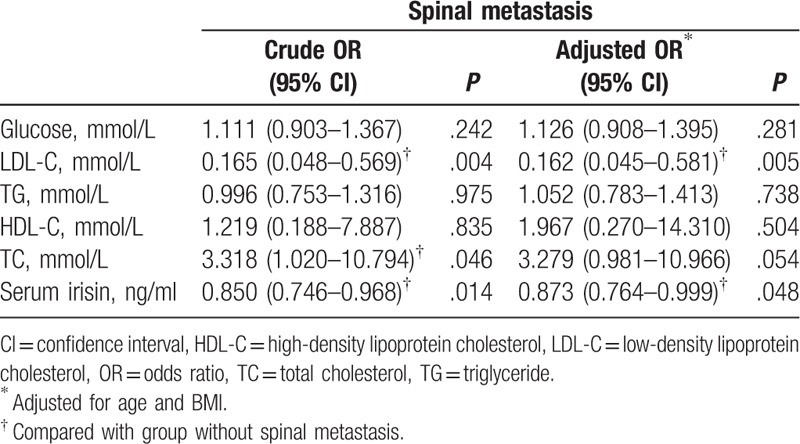
Crude and adjusted ORs for predictors of spinal metastasis.

### 3.3 Correlation between serum irisin with metabolic parameters, BMI, and the status of MetS

As irisin plays a role in the lipid metabolism and lipid metabolites may correlate to tumor progression, we analyzed the association between serum irisin and lipid metabolic parameters and BMI under the status of spinal metastasis. We used the Pearson correlation coefficient to identify the correlation between serum irisin and other parameters. We found that the serum irisin only had a significantly positive correlation with increased BMI (*r* = 0.263; *P* = .001). Furthermore, we assessed the correlation between the serum irisin and MetS status (0 = non-MetS; 1 = MetS) using the Spearman rank correlation coefficient; consequently, serum irisin was positively correlation with the presence of MetS (*r* = 0.207; *P* = .012) (Table 3).

**Table 3:**
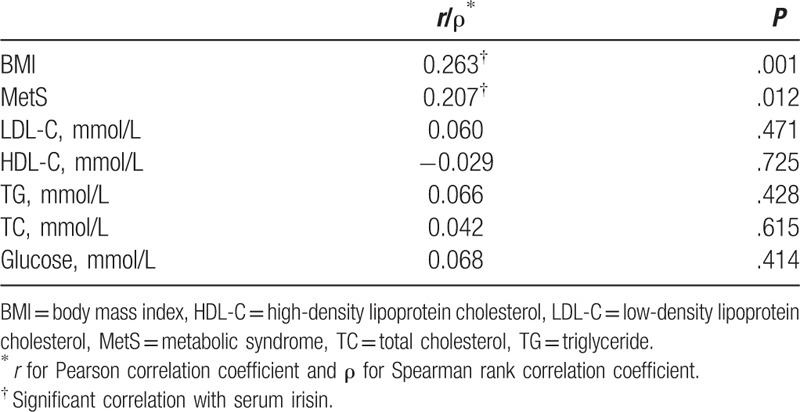
Analysis of correlation between serum irisin and lipid metabolic parameters and BMI.

### 3.4 The level of serum irisin in breast cancer patients with spinal metastasis or with MetS, respectively

**Figure 1:**
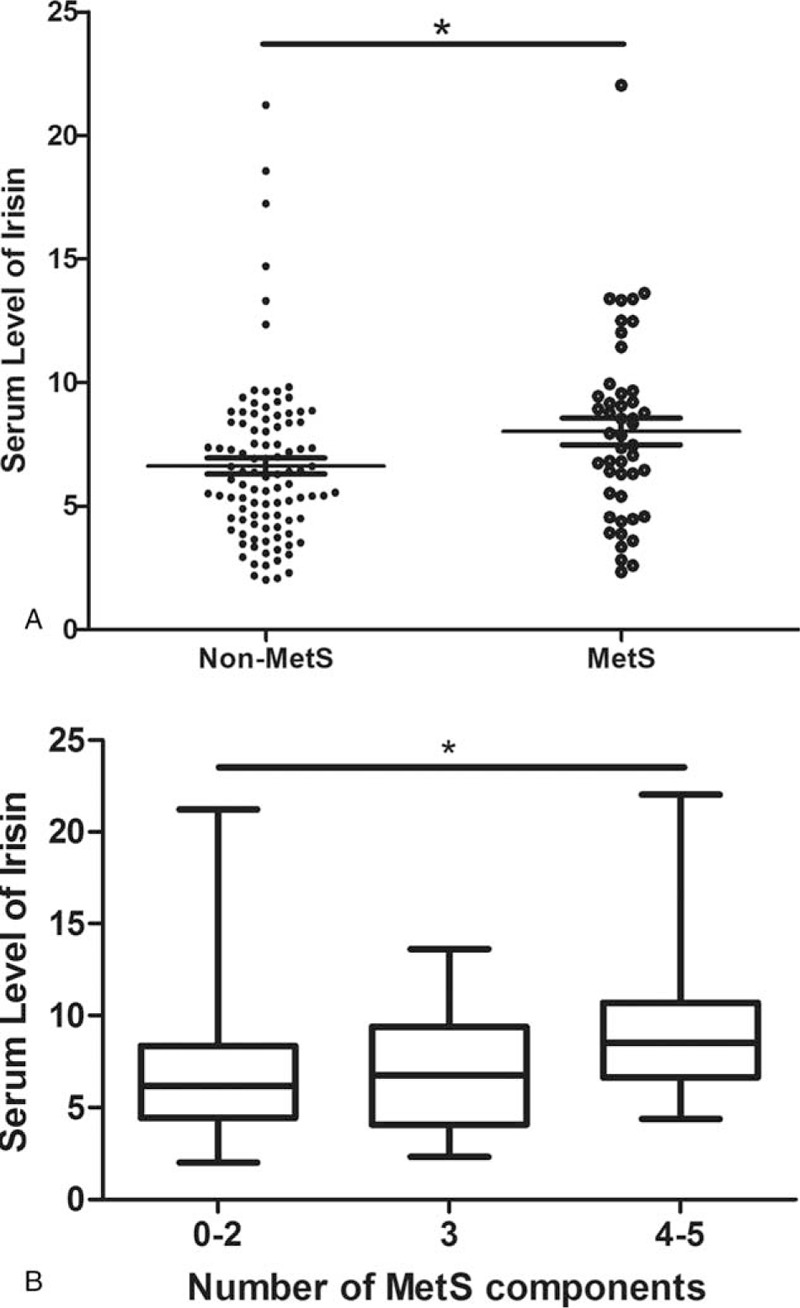
The level of serum irisin according to presence of MetS (A) or numbers of MetS components (B) in patients with breast cancer. Data are mean ± standard deviation. ∗∗*P* < .05. MetS = metabolic syndrome.

Figure 1A shows that the level of serum irisin tended to be lower in the breast cancer patients with spinal metastasis (*P* = .022). Figure 1B shows that with increasing numbers of MetS components, the serum level of irisin also increased (the ratios ± SD for 0–2 components vs. 3 components vs. 4–5 components were 6.59 ± 3.28 vs. 7.23 ± 3.57 vs. 9.16 ± 3.79, *P* = .008 between group 0–2 and group 4–5).

## 4 Discussion

The role of irisin in the progression of tumor remained controversial. One study found that irisin can suppress the migration, proliferation, and invasion of lung cancer cells in vivo^[22]^; on the contrary, other 2 studies found that irisin may promote the invasion and proliferation in human hepatocellular carcinoma.^[23,24]^ Nonetheless, Moon and Mantzoros^[25]^ found that irisin had no effect on cell proliferation and malignant potential in several cancer cell lines. These results implied the role of irisin in tumor may be tissue-specific. For breast cancer, Gannon et al^[26]^ found that irisin can suppress the malignant breast epithelial cell number. Furthermore, in one clinical research, the expression of serum irisin was found to be lower in patients with breast cancer compared with healthy people,^[15]^ indicating its protective role in breast cancer. However, its effect on breast cancer metastasis has not been fully investigated. In our study, the serum level of irisin was much lower in breast cancer patients with spinal metastasis than patients without spinal metastasis (Table 1). Furthermore, it showed to be an independent and protective factor in spinal metastasis after adjustments for age and BMI, the patients with higher serum level of irisin had a reduction of nearly 20% possibility to suffer from spinal metastasis (Table 2), indicating serum level of irisin protected the spine from breast cancer metastasis. In previous studies, irisin was found to prevent the bone loss, especially trabecular bone, in both animal experiments and clinical researches.^[27–30]^ Moreover, it can inhibit the osteoclast differentiating and promote osteoblast differentiating^[29]^ to maintain the bone quality, whereas the activation of osteoclast played a key role in the pathological process of bone metastasis.^[31]^ Considering favorable bone quality was important in resistance to metastasis of breast cancer, we deduced that the higher level of irisin played a protective role in the metastasis to spinal trabecular bone through improving the bone quality in this study.

Besides bone quality, irisin can also regulate the lipid metabolism.^[27,29,32]^ As metabolic disorders were closely associated with the status of breast cancer,^[33]^ we investigate the relationship between serum of irisin and MetS in the breast cancer patients. The serum level of irisin was much higher in patients with MetS and positively associated with the number of components of MetS (Fig. 1A and B), especially BMI (Table 3), although irisin was thought to conquer fat mass, it may be because of the presence of “irisin-resistance” and more irisin may be produced by the increased volume of adipose in patients with MetS or with higher BMI, which was supported by previous studies.^[34–36]^ Furthermore, when only focused on patients with MetS, the level of serum irisin was much higher in patients without metastasis to spine comparing with metastasis (Table 1); this result supported that the level of irisin was negatively associated with breast cancer to spinal metastasis.

Interestingly, the proportion of patients with MetS was smaller in group with metastasis, which seemed to be contrary to the results from previous studies, that metabolic disorders promote the invasion of breast cancer.^[37–41]^ In our analysis, this discrepancy may be caused by 2 reasons. First, we focused on the presence of spinal metastasis in patients with breast cancer but not between the patients and the healthy people and the volume in this study was relative small, so the proportion of MetS could be different with previous studies. Second, only the situation with spinal metastasis was investigated here, whereas breast cancer patients can suffer from other sites of metastasis; the effects of MetS in all metastatic situations were not fully investigated. In addition, unique bony metastasis has a better prognosis in patients with breast cancer comparing with other metastatic sites^[1]^; it was reasonable that the proportion of Mets in patients with bone metastasis was lower than patients without bone metastasis, as the latter participants may have other sites of metastasis and finally have worse prognosis.

There were some limitations to our study. First, it was a retrospective cross-sectional study with a small population, the causality between serum irisin and progression of breast cancer to bone metastasis should be further investigated. Second, spine-only metastasis was considered in this study and other sites of metastasis should be analyzed in further studies. Third, we recruited only participants with breast cancer; no healthy people were enrolled in the control group. Thus, a larger study with diverse populations should be performed to confirm the relationship between serum irisin and breast cancer to bone metastasis.

## 5 Conclusion

To our best knowledge, the role of serum irisin in breast cancer to bone metastasis was still not investigated. This study firstly did show that the serum irisin can be a candidate to predict the bone metastasis in patients with breast cancer, and the serum irisin was higher in MetS in patients with breast cancer. It was worth to further study in larger population.

## Acknowledgments

The authors acknowledge the assistance of investigators and all subjects for participants in this study.

## Author contributions

**Conceptualization:** Zheng-ping Zhang, Xue-fang Zhang, Ding-jun Hao.

**Data curation:** Zheng-ping Zhang, Hui Li, Tuan-jiang Liu, Ding-jun Hao.

**Formal analysis:** Zheng-ping Zhang, Xue-fang Zhang, Hui Li, Tuan-jiang Liu, Lin-hong Huang.

**Funding acquisition:** Zheng-ping Zhang.

**Writing – original draft:** Zheng-ping Zhang.

**Writing – review & editing:** Zheng-ping Zhang, Xue-fang Zhang.

**Investigation:** Hui Li, Tuan-jiang Liu, Ding-jun Hao.

**Software:** Hui Li, Tuan-jiang Liu, Li-ming He.

**Validation:** Hui Li, Tuan-jiang Liu, Qin-peng Zhao, Zi-jun Cao, Ding-jun Hao.

**Methodology:** Qin-peng Zhao, Ding-jun Hao.

**Project administration:** Ding-jun Hao.

**Supervision:** Ding-jun Hao.
